# Locus-specific expression analysis of transposable elements

**DOI:** 10.1093/bib/bbab417

**Published:** 2021-10-19

**Authors:** Robert Schwarz, Philipp Koch, Jeanne Wilbrandt, Steve Hoffmann

**Affiliations:** Computational Biology Group, Leibniz Institute on Aging - Fritz Lipmann Institute (FLI) Beutenbergstrasse 11, 07745 Jena, Germany; CF Life Science Computing, Leibniz Institute on Aging - Fritz Lipmann Institute (FLI) Beutenbergstrasse 11, 07745 Jena, Germany; CF Life Science Computing, Leibniz Institute on Aging - Fritz Lipmann Institute (FLI) Beutenbergstrasse 11, 07745 Jena, Germany; Computational Biology Group, Leibniz Institute on Aging - Fritz Lipmann Institute (FLI) Beutenbergstrasse 11, 07745 Jena, Germany

**Keywords:** RNA sequencing, transposable elements, tool comparison, simulation, differential expression analysis

## Abstract

Transposable elements (TEs) have been associated with many, frequently detrimental, biological roles. Consequently, the regulations of TEs, e.g. via DNA-methylation and histone modifications, are considered critical for maintaining genomic integrity and other functions. Still, the high-throughput study of TEs is usually limited to the family or consensus-sequence level because of alignment problems prompted by high-sequence similarities and short read lengths. To entirely comprehend the effects and reasons of TE expression, however, it is necessary to assess the TE expression at the level of individual instances. Our simulation study demonstrates that sequence similarities and short read lengths do not rule out the accurate assessment of (differential) expression of TEs at the instance-level. With only slight modifications to existing methods, TE expression analysis works surprisingly well for conventional paired-end sequencing data. We find that SalmonTE and Telescope can accurately tally a considerable amount of TE instances, allowing for differential expression recovery in model and non-model organisms.

## Introduction

The expression of transposable elements (TEs) has been repeatedly associated with various disorders including neurodegenerative [1, 2] and age-dependent diseases [3] or cancer [4, 5]. From an evolutionary perspective, however, expressed and reinserted TEs may play an advantageous role for the development of new genes by limiting gene conversion [6]. Likewise, it is suggested that TEs contribute to the heterogeneity and complexity of the brain [7]. While the activity of individual TEs is influenced by epigenomic factors such as DNA-methylation in vertebrates [8], a detailed understanding of the regulatory mechanisms is still missing. The major difference between TEs and other genomic features such as exons or lncRNAs is their high repetitiveness. Specifically, TE families contain long stretches of sequence that occur multiple times across the genome. Consequently, read aligners often face the challenge to correctly align TE reads to their locus of origin; i.e. the locus where the transcript read by the sequencer originated from. To deal with this multi-mapping read problem specialized tools have been developed in the past years.

The first important step to investigate this critically understudied part of genome regulation is the accurate and precise measurement of the expression of individual TE copies (TE instances). In this study, we systematically compare methods with regard to their ability to detect and quantify the expression of individual TE instances from simulated high-throughput sequencing data of three species (*Mus musculus*, house mouse; *Homo sapiens*, human; and *Nothobranchius furzeri*, turquoise killifish). Our analysis of the vertebrate model-organisms mice and human is complemented by the short-lived killifish *N. furzeri*, as it is quickly becoming an important model organism in aging research [9]. With an estimated TE content of 42.1%, its genome contains a considerable amount of TEs [10] and could be an interesting organism to study the regulation of TEs during aging. In contrast to the other two reference genomes used here, the assembly still is in a comparably early phase. Thus it provides TE expression benchmarks for genomes of lower quality.

**Table 1:** Overview of compared TE expression methods adapted from Lanciano et al. [31]. EM- Expectation maximization; TE- transposable element

Tool	Level	Used alignment tool	Multi-mapper handling	Used references	Ref.
SalmonTE	Family	Salmon	EM-Algorithm	Consensus of families	[13]
Telescope	Instance	Free Choice	EM-Algorithm	Reference genome	[12]
TEtranscripts	Family	Free Choice	EM-Algorithm	Reference genome	[15]
SQuIRE	Instance	STAR	EM-Algorithm	Reference genome	[11]
TEtools	Family	Bowtie/Bowtie2	Random assignment	TE pseudogenome	[14]

Major obstacles for TE detection and quantification are the technical read length limitation of most RNA sequencing (RNA-Seq) experiments and the high sequence similarity of TEs. Since most TEs are too long for many sequencers to be read at once or already underwent RNA processing prior to library construction, many reads are expected to map to multiple instances of a TE, i.e. a TE family. In addition, low quality genomes render the analysis of repetitive elements particularly hard, as TEs may be misplaced or absent in the reference. Therefore, the analysis of TE expression has frequently been restricted to TE families, which often means that a consensus sequence per family is calculated and used as a reference. Consequently, the detection and analysis of individual TEs with pathological or physiological relevance remains a critical challenge for the investigation of sizable parts of genomes across all kingdoms of life. Notably, family-level investigations are also obfuscated when family members are not coordinately up- or down-regulated. Only recently, tools such as SQuIRE [11] and Telescope [12] became available to tackle TE expression analysis on instance-level.

Here, we investigate to which extent existing methods implemented in SalmonTE [13], TEtools [14], TEtranscripts [15], SQuIRE and Telescope (see Table 1) can be used to quantify locus-specific TE expression. We simulated RNA-Seq data for *M. musculus*, *H. sapiens*, and the non-model organism *N. furzeri*, because as it allows benchmarking of tool performances. In contrast to real data, exact expression values and expression differences are known and thus serve as a gold standard in all evaluations. To this end, we modified the three methods originally designed for family-level analyses to obtain expression estimates for individual TEs. Using DESeq2 [16], a tool to estimate differential expression from count data of high-throughput sequencing reads, we additionally investigate the ability to recover differentially expressed TEs (DETEs) based on the tools’ alignments. Furthermore, our analysis provides insights into the relation of Kimura distances [17] and the ability to investigate expression levels of individual TE orders as defined by RepeatMasker [18]. In summary, our study provides a comprehensive assessment of the possibilities of DETE detection. This is an important step towards a better understanding of mechanisms underlying TE regulation in health, disease and aging.

## Methods

**Figure 1:**
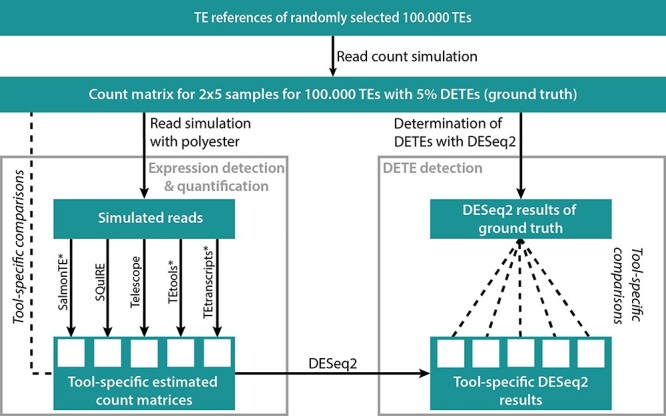
Workflow of tool evaluation. A count matrix for 100.000 randomly selected TEs was simulated, which was used to simulate reads with polyester. The tools SalmonTE^*^, SQuIRE, Telescope, TEtools^*^ and TEtranscripts (^*^ marks adapted tools) were applied to estimated counts per TE. The tool-specific estimated counts were compared with the ground truth (Expression detection & quantification). The ground truth of DETEs of the simulated TEs was determined with DESeq2 and compared to the tool-specific DESeq2 results (DETE detection). TE – Transposable element; DETEs – differentially expressed TEs.

The workflow of the tool evaluation is shown in Figure 1 and all in-house scripts, used in the following section, can be found at GitHub (simulation, evaluation and scripts: https://github.com/Hoffmann-Lab/TEdetectionEvaluation). Additionally, all command line calls are listed in Supplemental File 6.

### Generation of repeat reference library

We used the repeat annotation of RepeatMasker of *M. musculus* (mm10, based on Repeat Library 20140131 downloaded in January 2020, https://www.repeatmasker.org/genomes/mm10/RepeatMasker-rm405-db20140131/mm10.fa.align.gz), *H. sapiens* (hg38, based on Repeat Library 20140131 downloaded in January 2020, https://repeatmasker.org/genomes/hg38/RepeatMasker-rm405-db20140131/hg38.fa.align.gz) and *N. furzeri* (Nfu_20150522 downloaded in January 2020, https://nfingb.leibniz-fli.de/data/raw/notho4/Nfu_20150522.dispersed_repeats.Nf-RepLib.20141117.align.gz), along with the reference genome of *M. musculus* mm10 (v102 downloaded in January 2021 from ftp://ftp.ensembl.org/pub/release-102/fasta/mus_musculus/dna/), *H. sapiens* hg38 (v102 downloaded in January 2021 from ftp://ftp.ensembl.org/pub/release-102/fasta/homo_sapiens/dna/) and *N. furzeri* Nfu_20150522 (downloaded from https://nfingb.leibniz-fli.de/data/raw/notho4/Nfu_20150522.softmasked_genome.fa.gz) [10] to generate a reference sequence library of TEs in FASTA format for each organism. Specifically, coordinates of TEs from the RepeatMasker annotation were converted into BED format and used to generate a reference library of nucleotide sequences for each annotated TE by using bedtools getfasta (v2.29.2–41-g4ebba703) [19]. Genomic position, Kimura distance, strand and TE categories are tracked for each instance throughout the evaluation pipeline via unique TE identifiers (TE ids, in the format chr|start|end|TE-repclass|TE-family|TE-subfamily|score|KimuraDistance). All following steps are based on these generated reference libraries.

### Simulation of short read RNA-Seq data

In this study, we consider single-end (50 and 100 bp read length) as well as paired-end (100 bp read length) sequencing experiments. For either experimental setup, two distinct sets with five replicates each are generated. Throughout this study, the first set is considered a control (Set 1), while the second set contains 5% uniformly randomly drawn DETEs (Set 2; 2.5% up- and down-regulated, respectively). As a basis for our simulation, we uniformly randomly drew 100,000 TEs, i.e. LINE, SINE, LTR or DNA elements, with at least 100 bp in length and a known Kimura distance from the reference library.

Polyester (v1.22.0) from the Bioconductor universe (v3.10) [20] was used to simulate RNA-Seq data in FASTQ format. It allows simulating GC-biases and sequencing errors based on Illumina sequencing error profiles that are provided with the polyester package. A mean read coverage of 20-fold per TE was simulated and the fragment length for the paired-end data was drawn from a Gaussian distribution with a mean of 250 bp (SD = 25 bp; default settings, see Supplemental methods). The number of simulated reads per TE and sample is stored in a count matrix, which serves as a reference in the evaluation process. This matrix was also used as input for DESeq2 (v1.26.0), to identify those TEs that can be detected as differentially expressed with a perfect read assignment.

An additional simulation was done for *M. musculus* using an in-house script implementing an alternative GC-bias unaware simulation strategy using quality values of real experiments to introduce sequencing errors (see Supplement methods).

### Tool adaption, invocation and filtering of results

As described above, TEtools (v1.0.0), SalmonTE (v0.4) and TEtranscripts (v2.2.1) use different strategies to estimate TE expression at family-level (Table 1). We adapted the tools in order to evaluate their performance at the level of individual TEs instances and compare them with the dedicated instance-specific tools Telescope (v1.0.3) and SQuIRE (v0.9.9.92). As we did not change the algorithm of the tools, which are responsible for the assignment of multi-mapping reads, we do not expect an interfering of the outcomes.

By default, TEtools aligns reads to the instance-specific reference sequences and aggregates individual read counts afterwards to a family read count using a translation file. To suppress the aggregation step, we substituted the ids of TE families with ids of TE instances. Similarly, TEtranscripts uses an annotation file mapping TEs to their respective families. Again, we substituted the family names by TE ids to avoid the aggregation process. Since TEtranscripts and Telescope require precomputed alignments, simulated sequencing data was aligned with STAR (v2.7.6a) [21] according to the recommendation of TEtranscripts. Conversely, SalmonTE ships with an index for *M. musculus* and *H. sapiens* based on consensus sequences for each family. For our evaluation, we created an instance-specific index with Salmon (v0.9.1) [22] for each species instead, based on our repeat reference libraries. In the following, modified tools are referred to with an appended asterisk (^*^).

SQuIRE requires RepeatMasker’s ‘.out’ file format. To provide such a file, we translated the downloaded ‘.align’ files into the ‘.out’ format via an in-house script. This mapping is bijective, as the coordinates of each annotated TE are unique. From this, SQuIRE generates its own annotation file in BED format with SQuIRE-specific TE ids.

SQuIREs TE ids differ to ours, so that we cannot compare the results to the simulated counts by a simple merging process. However, both TE ids contain the genomic coordinates of the respective TE. These coordinates are unique for each TE and allow to find the corresponding instances in both count tables; i.e. there is a one-to-one relationship of the entries in the count tables.

Except for the modifications described above, all tools were run with default settings. Subsequently, the outputs were parsed and aggregated across all samples with an in-house script to obtain instance-specific read count tables for each tool, which were used for all downstream comparisons. We removed all TEs with 10 or less reads summed up over all 10 samples. This cut-off was chosen as it translates to more than one read per TE and sample on average. Removing low count genes allows the mean–variance relationship in the data to be estimated with greater reliability and also reduces the number of statistical tests that need to be carried out in downstream analyses looking at differential expression [23].

### Evaluation of the results

#### Expression detection and quantification

**Figure 2:**
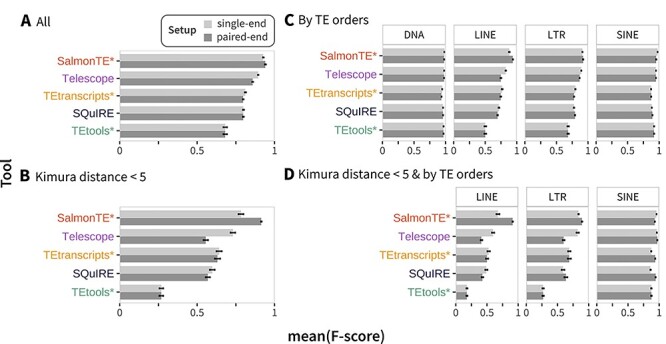
Comparison of TE expression detection in the *M. musculus* dataset. Mean F-scores were calculated across the ten replicates per tool and are given per setup (single-end 100 bp in light grey; paired-end 100 bp in dark grey) for (A) all TEs, (B) TEs with Kimura distance < 5, (C) orders of all TEs, and (D) orders of TEs with Kimura distance < 5. Note that in (D), DNA transposons are not shown, because no instance with a Kimura distance < 5 was present in the simulated data (see Supplemental File 1). TE — Transposable element.

Throughout this study, a TE is considered to be detected by a given tool in a particular replicate if the reported read count is equal to or larger than five. This step is common praxis to eliminate noise produced by occasional misalignments of individual reads [24]. Using this binary measure we are able to categorize the results for each TE as true positive (TP), false positive (FP), and false negative (FN). Using these, the recall (sensitivity), precision, and F-score are calculated. Additionally, mean F-scores were separately calculated for TEs grouped by Kimura distances (binned with step sizes of 5) and by TE orders. Both distances and orders are given by the RepeatMasker annotation (Figure 2).

**Figure 3:**
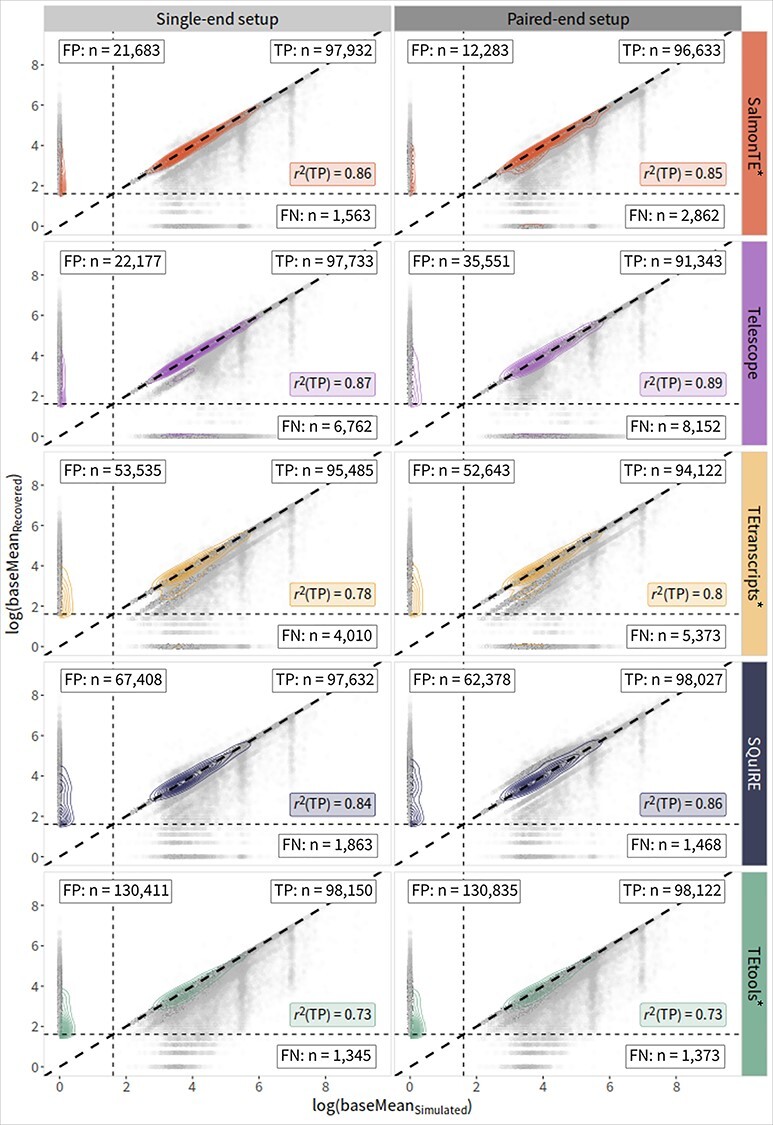
Comparison of recovered and simulated TE read counts of Set 2 of *M. musculus*. Scatter plots for simulated and recovered read counts for each tool (row) and sequencing setup (column; single-end: light-grey, paired-end: dark-grey). Dashed diagonal lines represent the perfect recovery (data points above: overestimation, points below: underestimation); dashed horizontal / vertical lines indicate the detection cut-offs to distinguish TP (upper right area), FP (upper left), and FN (lower right) at an expression value of 5. For each tool and setup, a coefficient of determination for TPs (r^2^(TP)) is given (colored boxes) as well as counts of TEs considered as TP, FP, and FN (boxes in respective areas). TNs are here filtered out due to their high number. Note that data points lying on the horizontal dashed line are counted to the upper categories (TP or FP) and those on the vertical are counted either to FN or TP, due to usage of the R-package ggpmisc (v0.4.0) [25]. FN — false negative; FP — false positive; TE—transposable element; TP—true positive.

Furthermore, based on the count data generated by each tool we calculated the mean expression levels per TE $i$ and Set

$$ {baseMean}_i=\frac{\sum_1^j{n}_{ij}}{j} $$,

where ${n}_{ij}$ (Read counts $\in{K}^{ixj}$) is the count of TE $i$ in replicate $j$ and compare them to the mean expression levels of the simulated TEs. Based on these base means, the coefficient of determination (r^2^) was calculated from simulated and recovered read counts for true positives only (r^2^(TP)). For visualization (Figure 3, Supplemental Figs 4–7), the logarithm of the mean expression levels was calculated and set to 0 if the original value was 0.

### DETE detection

DETEs generated in our simulation may escape the detection by DESeq2 due to low expression, low expression fold changes and/or high dispersion. Additionally, DESeq2 might identify DETEs that were not simulated as such. To distill the set of DETEs that are detected by DESeq2 using the counts of an ideal aligner, we first ran DESeq2 on the simulated counts directly. Using the DESeq2 output, the subset of TEs detected as differentially expressed was used for further analysis as our ground truth. In this setup, a perfect aligner would have the power of 1. Subsequently, we ran DESeq2 with the count tables generated by all tools tested in this study. Afterwards, we evaluated the tools by comparing the output to the ground truth. The evaluation is based on true positive rates (TPRs) and the false discovery rates (FDRs) and is calculated as follows: (1) sort the DESeq2 result table in ascending order by the adjusted p-value; (2) count TPs and FPs in a cumulative manner; (3) use the cumulative values to calculate a TPR and FDR for each instance.

### Ranking

The tools are ranked for each part of the evaluation (detection and quantification of TE expression, detection of differential TE expression), based on different categories within the evaluation parts (see Supplemental methods).

## Results

The following results are based on the 100-bp polyester-based data sets, if not stated explicitly otherwise. The results of a complementary alternative simulation are shown in the Supplementary Material.

### Simulation

After filtering for minimum read count (see Methods), 99 427 simulated expressed TEs were used for downstream analyses of *M. musculus*, 99 765 of *H. sapiens* and 99 235 of *N. furzeri* (Supplemental File 1). DESeq2 predicted 5 153 differentially expressed TEs in the *M. musculus* dataset (adjusted *p*-value threshold of 0.05), 5 174 in *H. sapiens* and 5 148 in *N. furzeri* when the simulated counts are used directly. These sets of DETEs are used as ‘ground truth’ of each species (see Methods).

### Detection of TE expression

We first analyzed the tools’ abilities to distinguish between truly expressed and silent TEs. Overall, similar observations can be made in *M. musculus* (Figure 2), *H. sapiens* and *N. furzeri* (Supplemental Figure 3). Across all species and sequencing setups, our results consistently indicate that tools using expectation maximization algorithms to assign multi-mapping reads perform better on average than TEtools^*^, which omits such a step. The overall improvement upon using paired-end data, as measured by the F-score (Supplemental File 2), appears to be surprisingly limited in all species when considering TEs across all Kimura distances (Figure 2A; Supplemental Figure 3A). With median F-scores from 0.93 (single-end) to 0.97 (paired-end) only SalmonTE^*^ shows consistently improved F-scores across all species. In some cases, the single-end data delivers higher F-scores compared to paired-end data, e.g. Telescope for *M. musculus* (0.86 to 0.89, Figure 2A).

Consequently, the most substantial F-score increase comparing single-end (0.78) and paired-end (0.91) is observed for SalmonTE^*^ for Kimura distances <5 in *M. musculus*. On the other hand, the F-score is significantly decreased for Telescope for the same set of TEs from 0.73 to 0.56 (Figure 2B). Tools using the STAR aligner (Telescope, TEtranscripts^*^, and SQuIRE) obtain higher F-scores for single-end than for paired-end data in *M. musculus*. However, in *H. sapiens* and *N. furzeri*, SQuIRE and TEtranscripts^*^ show the expected improvement of F-scores using paired-ends for Kimura distances <5 (Supplemental Figure 3B).

Conversely, the length of single-end reads had a stronger impact. Compared to 50 bp single-end reads, the mean F-scores for the 100 bp single-ends improved from 0.8 to 0.82 across all tools in *M. musculus* (0.87 to 0.91 in *H. sapiens*, 0.76 to 0.82 in *N. furzeri*).

When considering F-scores for the four investigated TE classes (DNA, LINE, LTR and SINE) separately, best results are consistently obtained for DNA elements (Figure 2C, Supplemental Figure 3C). Despite its large number of DNA elements with a Kimura distance <5 (*n* = 10 916), this is also true in *N. furzeri*. On the other hand, the lowest F-scores are observed for LINEs with a Kimura distance <5 in all species (Figure 2D; Supplemental Figure 3D, Supplemental File 2). Again, we also observe the strongest F-score increase for LINEs upon paired-end data usage for SalmonTE^*^ in all species (from 0.67 to 0.91 in *M. musculus*, from 0.90 to 0.98 in *H. sapiens* and from 0.83 to 0.93 in *N. furzeri*).

The superior performance of SalmonTE^*^ is also confirmed using the alternative simulation strategy. Importantly, the ranking of all tools is comparable using this alternative data, only SQuIRE and TEtranscripts^*^ swap their ranks (see Supplemental File 5). Here, however, the tools appear to make slightly better use of paired-end information.

### Quantification of TE expression

In terms of the tools’ performances in quantifying TE expression, we evaluated the expression detection performance based on FP, TP, and FN counts, as well as r^2^(TP), for single- and paired-end data. Results for *M. musculus* are shown in (Figure 3). Analogous data for the other species and simulations are shown in the Supplement (Supplementary Figs 4–7; Supplementary File 3). SalmonTE^*^ and Telescope continuously show the lowest counts of FPs across all studied species and setups ranging from 3 028 in *H. sapiens* (SalmonTE) to 35 551 in *M. musculus* (Telescope). Surprisingly, in the case of Telescope, the numbers of FPs are consistently increase by using paired-end data. The differences between the tools are less pronounced regarding FNs. Here, TEtools^*^ consistently yields the lowest count of FNs across all species and sequencing setups.

We observe a tendency of SalmonTE^*^, TEtranscripts^*^ and TEtools^*^ towards underestimating the TP counts. This is most pronounced in *N. furzeri* (Supplementary Figure 6) where almost half of simulated TEs (48%; median of all three tools) receive fewer reads than simulated while this is the case for only 24% of human TEs. Overestimation of TE expression appears to be most pronounced for TEs quantified with SQuIRE, which can be consistently observed in all species and sequencing setups.

Our analysis also indicates that the r^2^(TP) values obtained with Telescope are the only ones consistently improving when paired-end data is used, while the other tools exhibit inconsistencies or, in the case of TEtools^*^, don’t improve. The majority of the tools show slightly increased r^2^(TP) for *M. musculus* and *N. furzeri*, and slightly decreased values in *H. sapiens*.

### Differential TE expression

**Figure 4:**
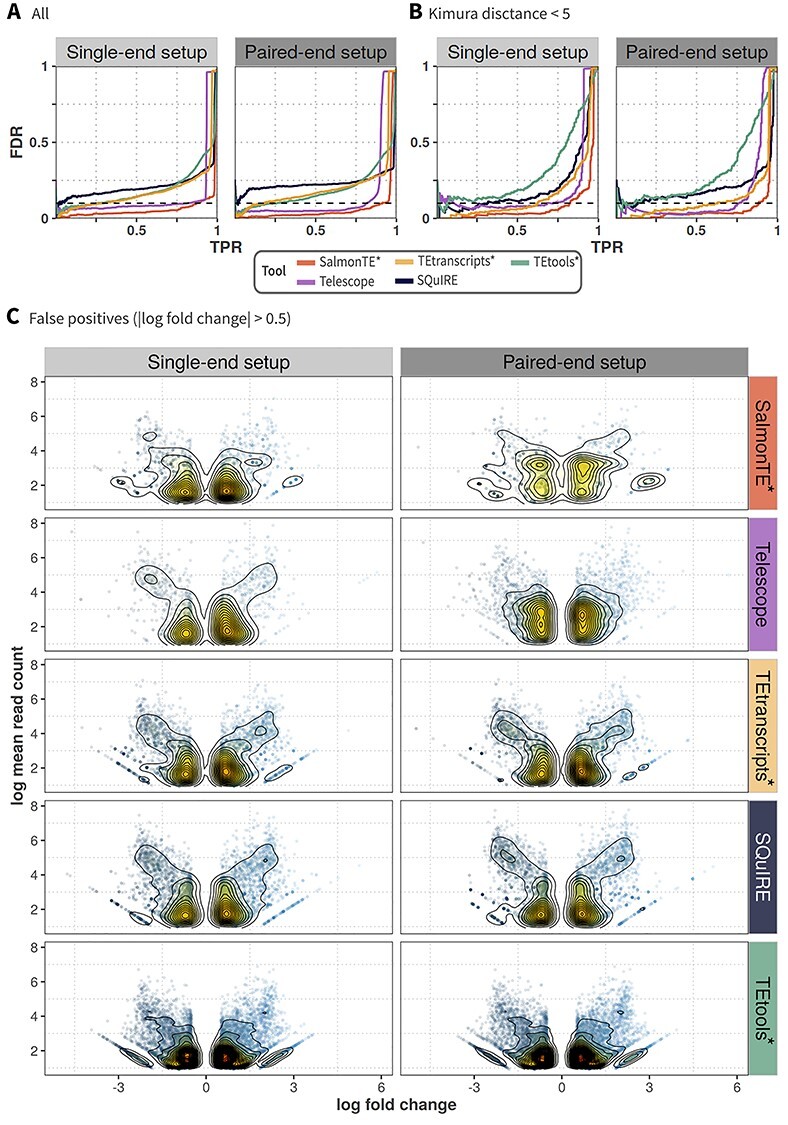
Comparisons of DETE detection performance and expression changes in all FPs of Set 2 versus Set 1 for single-end (left column in respective panel, light-grey) and paired-end setup (right column, dark-grey). (A, B) DETE detection performance (recovery of TEs simulated as differentially expressed in Set 2 compared to their expression in Set 1) is visualized as TPR in relation to FDR, shown per tool (lines) for (A) all detected TEs and (B) TEs with Kimura distance <5. The dashed horizontal lines represent a fixed FDR of 0.1. (C) Expression fold changes of FPs between Set 2 and 1 in contrast to mean read counts across all replicates for each tool (rows). Data points with a |log(fold change)| < 0.5 were removed for the sake of clarity. DETE – differentially expressed TE; FDR – false discovery rate; FP – false positive; TE transposable element; TPR – true positive rate.

Subsequently, we evaluated the ability to detect expression changes with DESeq2 based on the tools’ read count tables. For benchmarking, we used the FDRs and TPRs to analyze the DETE detection performances (Figure 4A and B; Supplemental Figure 8). Exact numbers for the recall are given in Supplemental File 4. In general, we observe that the ranking of the tools in this exercise is comparable for all genomes, sequencing- and simulation strategies.

At a fixed FDR of 0.1, SalmonTE^*^ achieves the highest TPRs (0.81 to 0.99) across all data sets. With TPRs from 0.47 to 0.95, Telescope always takes the second rank. Both tools benefit from paired-end information. Conversely, TPRs across all data sets for TEtranscripts^*^ (0.26 to 0.43) or TEtools^*^ (0.24 to 0.61) are smaller and results do not substantially improve with paired-end reads. SQuIRE does not reach TPRs bigger than 0.05 for an FDR of 0.1 in all species and simulation strategies.

Overall performances are apparently impacted by the genome quality. Consequently, results in *H. sapiens* are generally better compared to *M. musculus* and *N. furzeri*. With the exception of individual performances for paired-end data, the results for *M. musculus* are generally better than for the non-model genome of *N. furzeri*. Especially Telescope shows a decline in performance when applied to the killifish transcriptome simulation. Again, for all species investigated here, SalmonTE^*^ outperforms the other tools (cf. Supplemental Figure 8).

Calculating TPRs for TEs with a Kimura distance <5 (Figure 4B, Supplemental Figure 8), we observe that SalmonTE^*^ and Telescope maintain their leading ranks. Again, paired-end data typically improves the results of both tools. The TPRs of SalmonTE^*^ (0.73 to 0.97) and Telescope (0.70 to 0.98) indicate their overall suitability for the expression measurement of young elements in both, model and non-model organisms.

Given that SQuIRE ranks overall second in the quantification of TE expression, it is surprising that the tool shows a comparatively poor performance in the differential expression exercise (TPR of 0.002 to 0.02). This result may be explained by the combination of a relatively high number of FPs and a stronger tendency for over-estimation of read counts (Figure 3; Supplemental File 3). To investigate the role of FPs in this phenomenon, we selected all TEs that were simultaneously wrongly detected in Set 1 and Set 2. This examination revealed populations of 1 571 and 1 518 TEs in the single- and paired-end setups, respectively, with comparably high read counts (mean count >20) and fold-changes |log(fold change)| >1, Figure 4C). Of these, 97% were in fact also wrongly detected as differentially expressed. Thus, we reason that the rather large number of FPs in combination with more pronounced mis-estimations of read counts could explain this result.

## Discussion

While TEs have repeatedly been shown to play a role in pathological and physiological processes [3, 4, 26, 27], little is known about their expression patterns across different species, tissues and developmental stages. As the elevated expression of TEs has been observed during aging, a better understanding of molecular causes and consequences of TE dysregulation could, for instance, also yield new insights in age-related diseases and phenotypes [3, 28–30]. The lack of knowledge on TE regulation may be a consequence of a perceived lack of suitable methods to investigate the expression of repetitive regions of the genomes. Analyses on the level of TE families may only reveal transcriptional variation of single instances or sets of them if the changes are strong and consistent enough to compensate for contra-directional expression patterns of other family members. This may be exceptionally critical for families with multiple active instances. The most important shortcoming of family-level strategy, however, is the blindness regarding the precise genomic context in which the expression occurs. Since it is hard to imagine that all active instances of a TE family are governed by the same mechanisms or exert identical effects on cellular functions, it is critical to investigate TE expression at the level of single instances.

While achieving this goal is hampered by inherently high degrees of sequence similarity, technical, and, ultimately, financial limitations, our study explores to which extent the measurement of locus-specific TE expression is achievable with existing methods. Notably, three of the tools tested here are originally designed to work on the family-level only (SalmonTE, TEtranscripts and TEtools).

### Detection of expression

The analysis of repetitive elements is critically obfuscated by multi-mapping reads and different strategies have been devised to assign these reads over the years [31]. Two of the methods tested here, implemented by Telescope and SalmonTE, involve read-generating models and maximum likelihood objectives for distributing multi-mapping reads to candidate loci. Of note, SalmonTE is based on Salmon and relies entirely on its quasi-read-mapping algorithm. Different solutions, also involving expectation maximization algorithms for the assignment of multi-mapping reads, are employed by SQuIRE and TEtranscripts. TEtools, also intended for use on the family-level only, omits such a step and assigns multi-mapping reads randomly to the TE pseudogenome (Table 1).

In general, we observe that the detection of expressed TEs works better with tools that employ expectation maximization steps, i.e. SalmonTE^*^, Telescope, TEtranscripts^*^ and SQuIRE (Figure 2A). Telescope and TEtranscripts^*^ work with pre-computed alignment files and recommended alignment parameters are the same for both tools. Even though Telescope and TEtranscripts^*^ were thus called with the very same alignment files, their performances differed strongly. Thus, it is safe to assume that these differences are due to post-alignment calculations rather than the accurate assignment of reads to a genomic locus by the aligner. Apparently, SQuIRE’s strategy to assign reads to multiple loci (Supplemental File 1) tends to increase the number of falsely detected expressed TEs. In turn, this has negative effects on the F-score statistics.

The analysis of repetitive genomic regions is substantially influenced by the amount of effective sequence information. Thus, paired-end setups should facilitate the detection of transcripts from many TEs [32]. In general, SalmonTE^*^ is able to benefit the most from the additional sequence information in paired-end data. However, the degree to which individual tools take advantage of the additional sequence information varies strongly. Surprisingly, in the case of Telescope, paired-end data led to a drop of performance in detecting expressed TEs in all genomes and simulation strategies. This phenomenon might in part be explained by the tool’s filtering strategies. By default, reads and read-pairs mapping to more than 100 possible loci are removed. In comparison with single-end, paired-end data typically reduces the number of multi-mappers such that fewer reads are removed by this filter [32]. Consequently, a higher number of read alignments are reported (shown by increased mapping rate, Supplemental File 1). On the flip side, the threshold might also substantially safeguard against misalignments and could explain the elevated number of FPs for paired-end data.

The Kimura distance [17] of a TE describes the sequence similarity to its family consensus sequence. Since sequence similarity plays a crucial role in tool performances, we evaluated the tools for distinct Kimura distances. As expected, we observe decreasing F-scores for elements with low Kimura distances (Figure 2B, Supplemental Figure 2), which can be mitigated by paired-end sequencing strategies. Naturally, this has consequences for exact measurement of elements from active families. Among young elements with a high sequence similarity (Kimura distance <5), LINE instances of *M. musculus* are especially difficult to track, as their similarity distribution is skewed to a Kimura distance of 0 (Supplemental Figure 1). In contrast to DNA transposons, families of LINE, SINE and LTR classes are still active in *M. musculus* [33]. The detection of young LINE instances appears to be more successful in *H. sapiens* and *N. furzeri*, since in these genomes the distribution of Kimura distances is not as strongly skewed to 0 indicating a reduced or less recent LINE activity (Supplemental Figure 1). On the other hand, all tools perform well for DNA transposons. In the case of *N. furzeri* this is a bit surprising, as this organism appears to have a very high number of young DNA transposons. Here, the cut-and-paste transposition mechanism of DNA transposons and rather small family sizes [34] appear to substantially ameliorate the multi-mapping read problem and its consequences.

While SalmonTE^*^ came up as the top runner in most of our benchmarks, we noted some exceptions. Importantly, it did not recover the highest number of ‘truly’ expressed TEs (TP). This might be a drawback in all such cases where maximum sensitivity is of essence. Furthermore, SalmonTE^*^ does not show the highest *r*^2^ values for the count estimation of TP, as the underestimation of the counts is more pronounced compared to other tools (Supplementary File 3).

### Quantification and detection of differential expression

In light of mounting evidence for the biological relevance of TEs in health and disease, we evaluated the applicability of the five methods for differential expression analysis. A critical factor for the reliable detection of differential expression is the accuracy of read count estimates. While the majority of the tools show a systematic bias, i.e. an underestimation, in single- and paired-end setups, paired data improves estimates on average (Figure 3, Supplemental Figures 4–7). This result can be expected as the number of unaligned or misaligned reads is reduced by additional paired-end information. Despite the fact that Telescope yields an increased number of FPs when paired-end data are used, it is able to substantially improve the read count estimates for truly expressed TEs, and shows the highest accuracy and precision (i.e. in *M. musculus*, Figure 3). The best performance in terms of detecting DETEs is observed for SalmonTE^*^.

On the flip side, SQuIRE’s usability for the detection of DETEs appears to be limited by the assignment of reads to multiple loci (Supplemental File 1). Despite the second rank considering the quantification of TE expression (Table 2), a substantial number of FPs show such a high difference between Set 1 and Set 2 (Figure 4C) that they are called as DETEs. Consequently, the TPR for an FDR of 0.1 of SQuIRE lags behind the other evaluated approaches in this specific exercise.

**Table 2:** Ranking of the tools concerning their performance of detection and quantification of TE expression and detection of differential expression. TE- transposable element

Tool	Detection of TE expression	Quantification of TE expression	Detection of differential TE expression
SalmonTE^*^	1	1	1
Telescope	2	4	2
SQuIRE	3	2	5
TEtools^*^	5	3	3
TEtranscripts^*^	4	5	4

### Simulation

Simulations allow the systematic analysis of computational methods when the ground truth for actual data is unknown or difficult to obtain. On the flip side, simulated data cannot reflect reality in all its facets. For instance, unknown alternative transcription starts, termination sites, or post-transcriptional processes leading to RNA degradation lead to specific transcripts not covered by any annotation. Thus, simulations may not reach the level of complexity in real data. Also, it is essential to keep in mind that models and parameters accounting for phenomena such as GC-biases or sequencing errors are global approximations. However, for benchmarking alignment algorithms entirely relying on the sequence information of reference genomes and individual reads or read-pairs, such simulations provide indispensable insight into the tools’ capabilities to deal with repetitive sequences.

## Conclusion

Within the limits of our simulation study, a tool originally designed for family-level quantification, SalmonTE, emerges as the most convincing results. In addition to favorable results in detecting expressed TEs, SalmonTE^*^ results enable a surprisingly high recall of differentially expressed TE transcripts. The general ranking of the tools regarding DETE detection (Table 2) based on TPRs for an FDR of 0.1 — SalmonTE^*^ performing best, Telescope second, TEtools^*^ third, followed by TEtranscripts^*^ and SQuIRE — holds for all sequencing setups and studied species.

Arguably, the detection, quantification, and differential expression analysis of transcribed TEs remains one of the most challenging tasks in genome research. The misplacement or absence of instances from reference genomes, especially in the case of active TEs, insufficient read lengths, and high degrees of sequence similarity often restrain investigations of this biologically relevant class of RNA. Despite all technical difficulties, our analysis shows that an accurate and precise reference mapping of many individual TEs is already possible and encourages a more intensive research into this direction.

**Key Points**

- Accurate expression assessment of individual transposable elements is possible and can help to study their biological role more in detail, here demonstrated on simulated data.
- RNA-Seq protocols affect the detection of locus specific TE expression, however, even older protocols, e.g. single-end, are appropriate to get a comprehensive overview about individual TE expression.
- Detection of differentially expressed TE instances can be achieved with existing methods, partially with slight modifications.

## Supplementary Material

figure_S1_bbab417

figure_S2_bbab417

figure_S3_bbab417

figure_S4_bbab417

figure_S5_bbab417

figure_S6_bbab417

figure_S7_bbab417

figure_S8_bbab417

Supplemental_File_1_bbab417

Supplemental_File_2_bbab417

Supplemental_File_3_bbab417

Supplemental_File_4_bbab417

Supplemental_File_5_bbab417

Supplemental_File_6_bbab417

2021-05-20_Supplemental_Methods_bbab417

## Data and materials availability

All in-house scripts, used in this study, can be found at GitHub (simulation, evaluation, and scripts: https://github.com/Hoffmann-Lab/TEdetectionEvaluation) and all command line calls are listed in Supplemental File 6. Data including references of transposable elements, simulated FASTQ files, and count tables of the tools are available at https://zenodo./record/4561751 [35]. The used FASTQ files for the alternative simulation are publicly available on GEO (single-end: GSM1944865, paired-end: GSM1716945) [36, 37].

## Notes

**Robert Schwarz** is a PhD candidate in Bioinformatics at Friedrich-Schiller-University Jena, Germany, and is based at the Leibniz Institute on Aging—Fritz Lipmann Institute (FLI). His research focuses on the characterization of transposable elements and its activity.

**Philipp Koch** received his doctorate from the University of Jena in 2016. Since then, he has been working within the Core Facility Life Science Computing at the FLI on service-oriented bioinformatics analyses and on the development of software tools for molecular genetics.

**Jeanne Wilbrandt** received her doctorate in Biology from the University of Bonn in 2018. Her research focused on genomics and bioinformatics approaches. Currently, she works as Data Steward within the Core Facility Life Science Computing at the FLI.

**Steve Hoffmann** is group leader at the FLI and professor for Computational Biology at the Friedrich Schiller University Jena. His main research interest is the development of methods to investigate the regulation of genome activity.
