# Supplementary figures and images for: Locus-specific expression analysis of transposable elements
Source: Brief Bioinform. 2021 Oct 19;23(1):bbab417. doi: 10.1093/bib/bbab417 (PMC8769692; doi:10.1093/bib/bbab417)

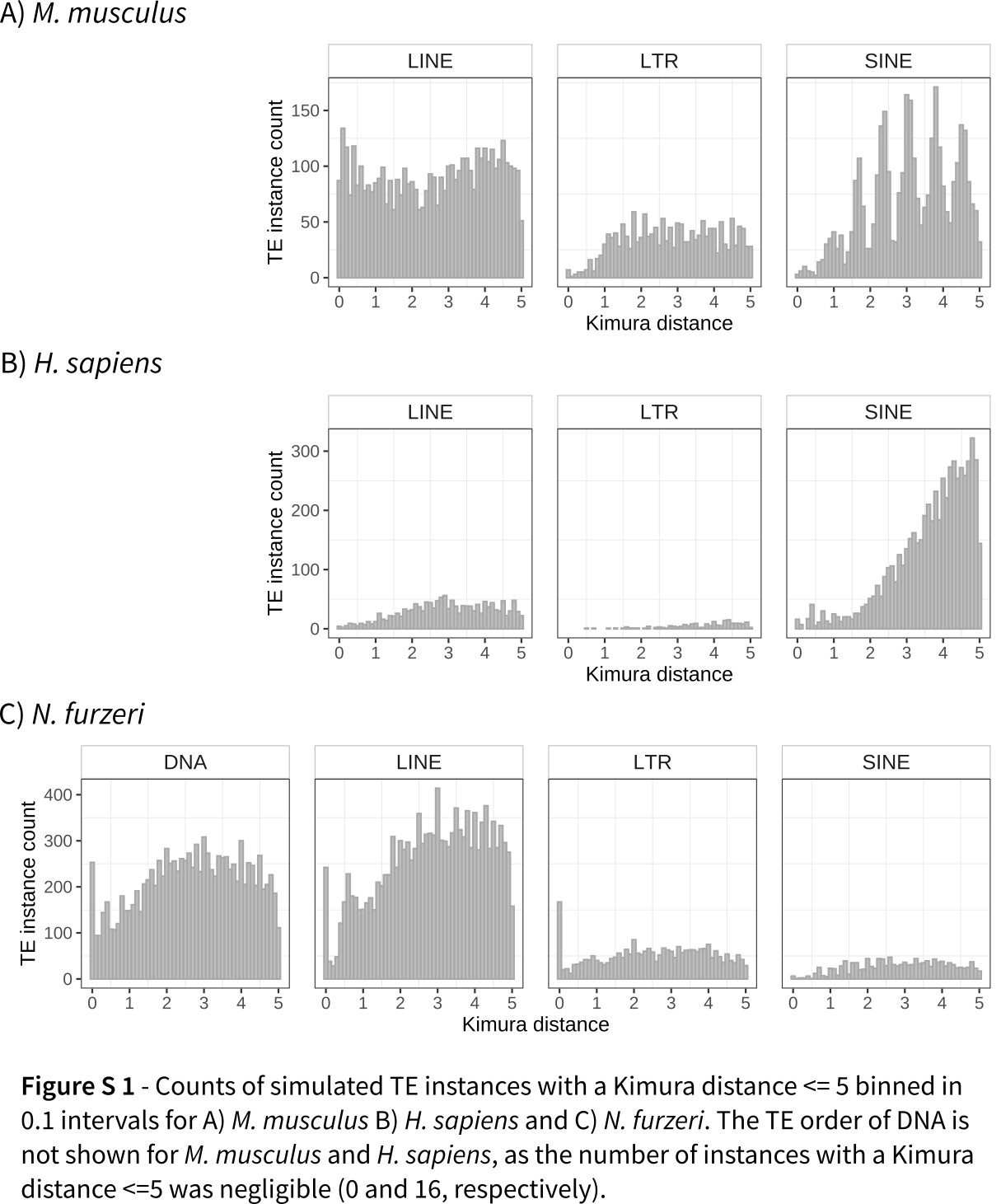

Supplement: figure_S1_bbab417 [file figure_s1_bbab417.jpeg]

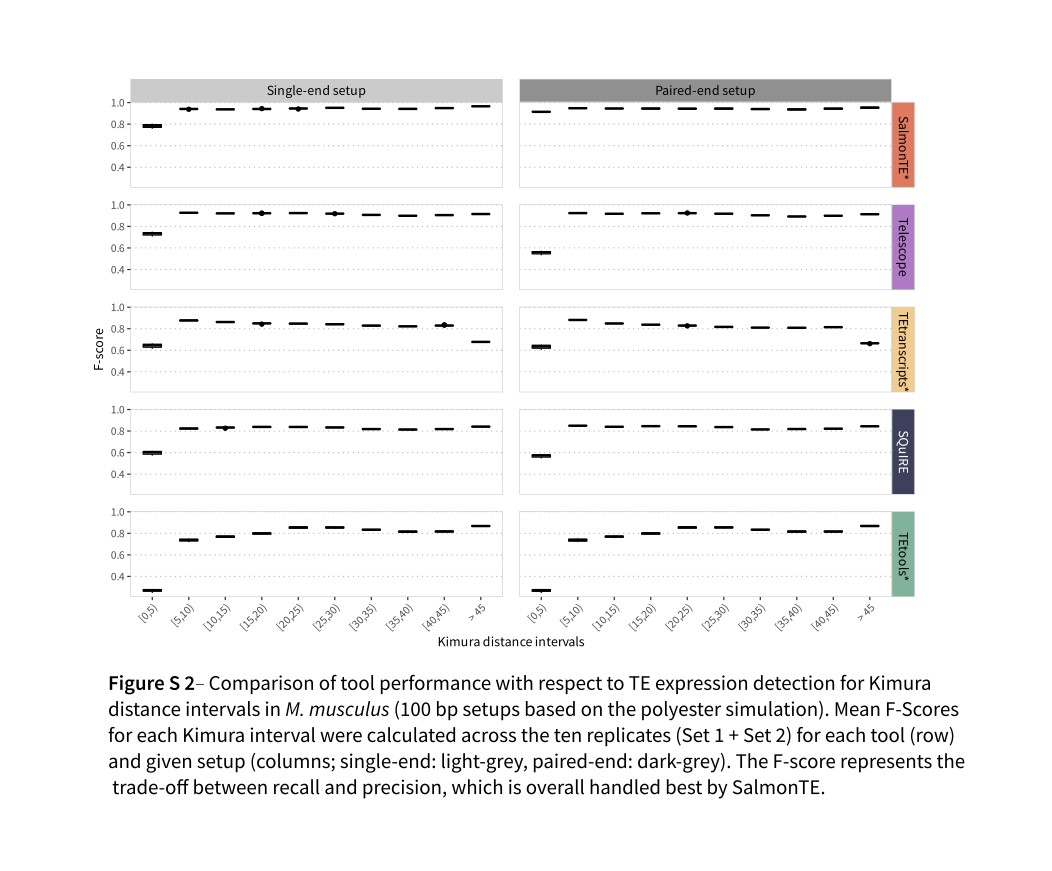

Supplement: figure_S2_bbab417 [file figure_s2_bbab417.jpeg]

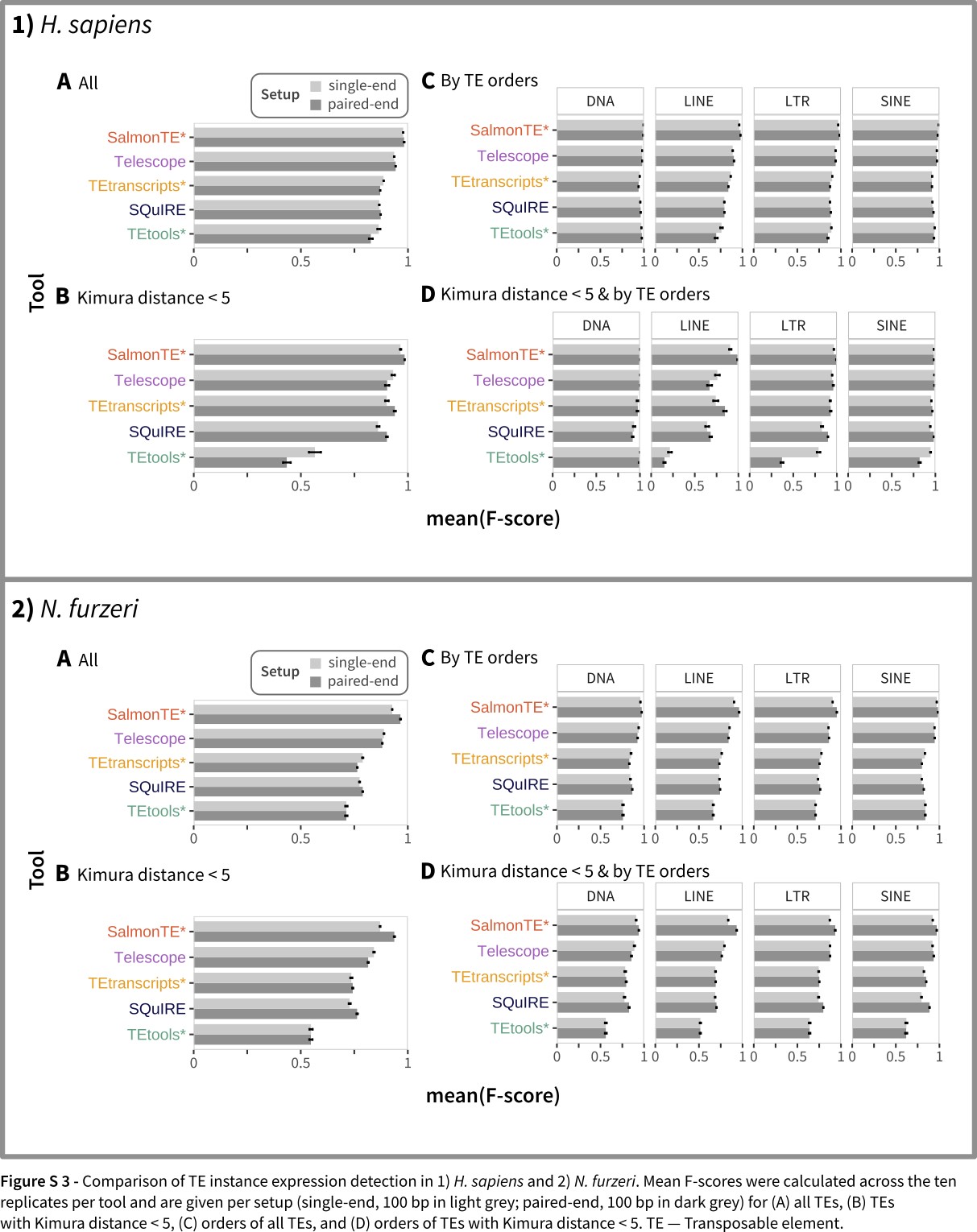

Supplement: figure_S3_bbab417 [file figure_s3_bbab417.jpeg]

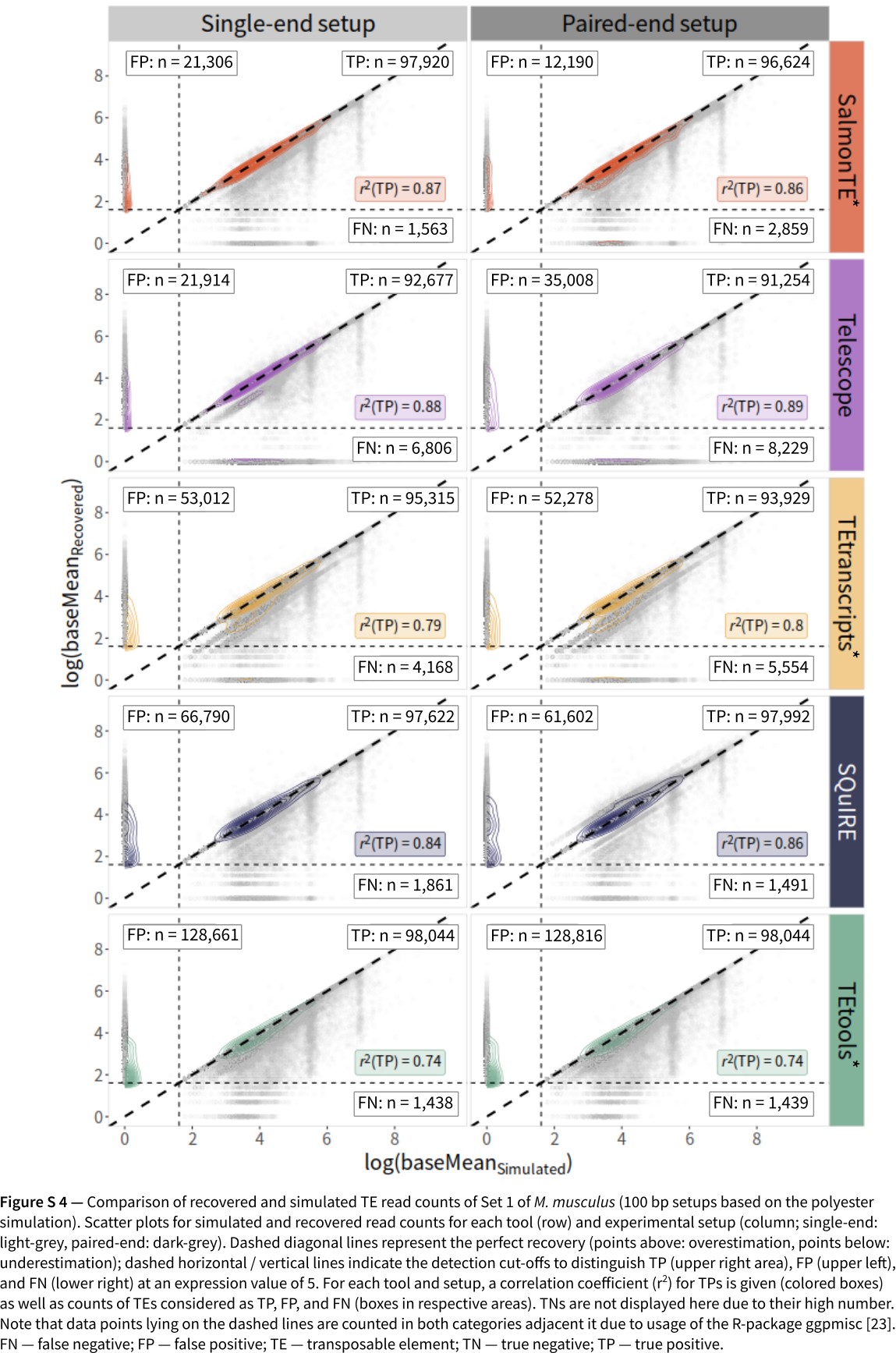

Supplement: figure_S4_bbab417 [file figure_s4_bbab417.jpeg]

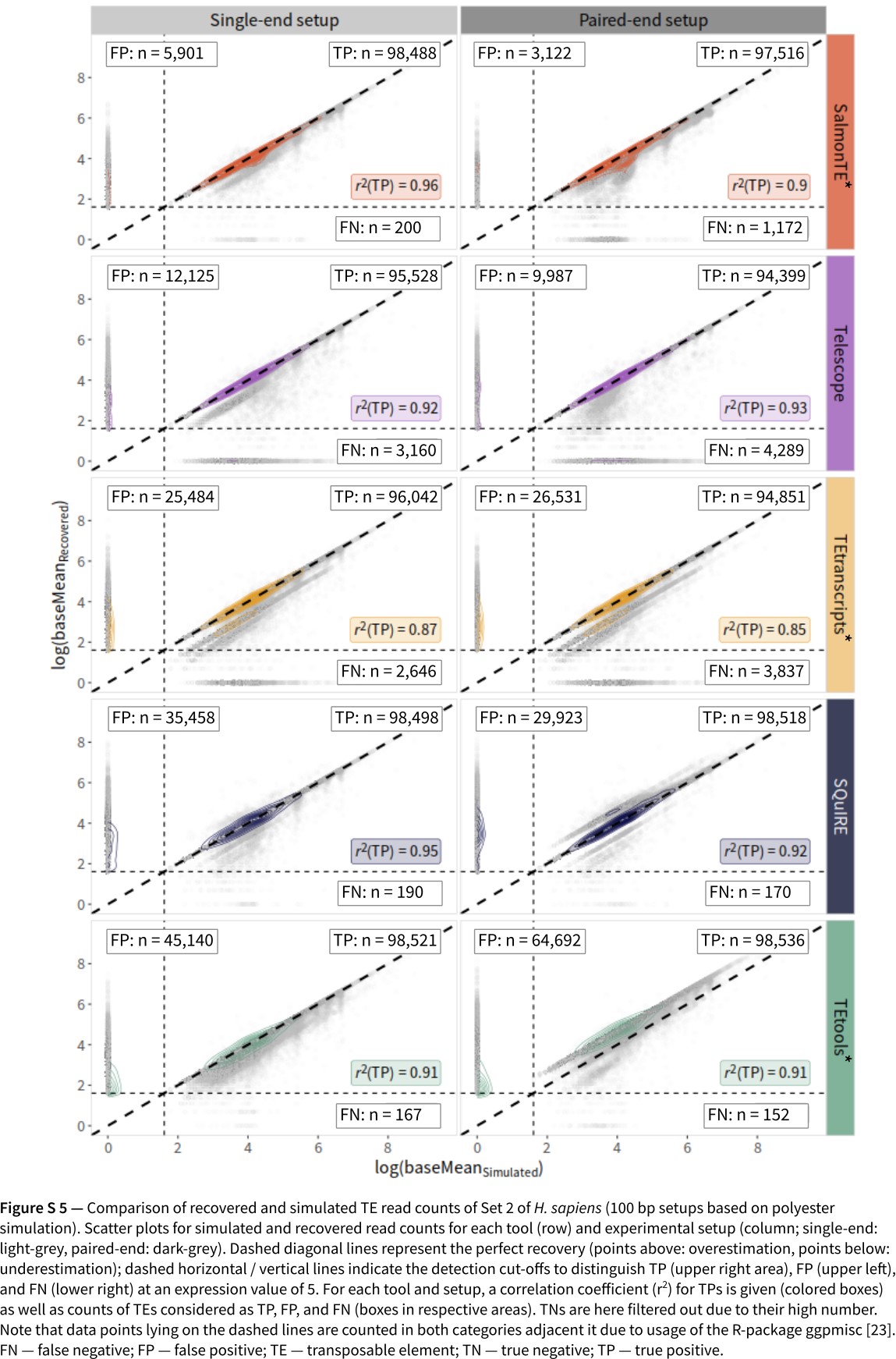

Supplement: figure_S5_bbab417 [file figure_s5_bbab417.jpeg]

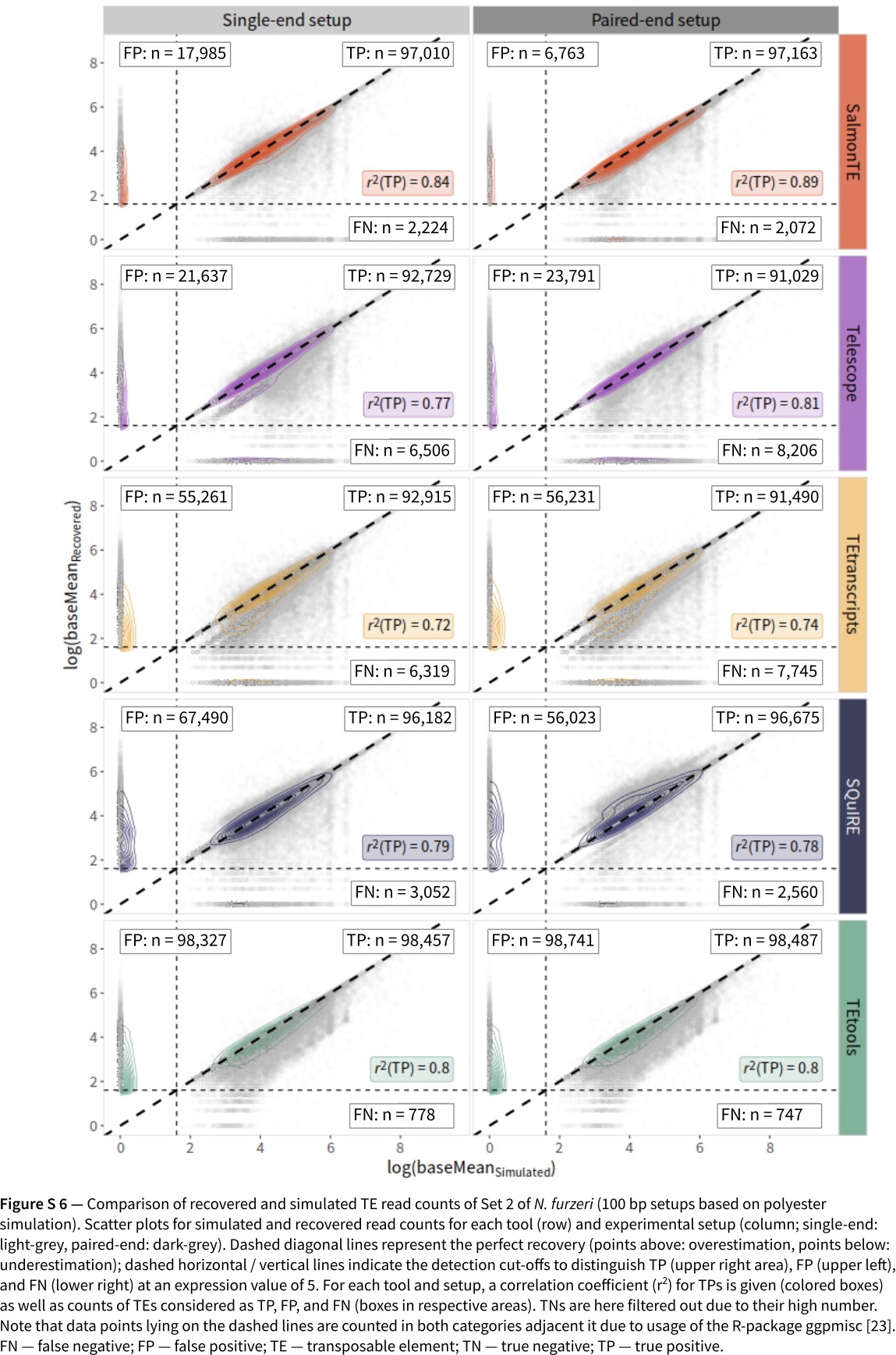

Supplement: figure_S6_bbab417 [file figure_s6_bbab417.jpeg]

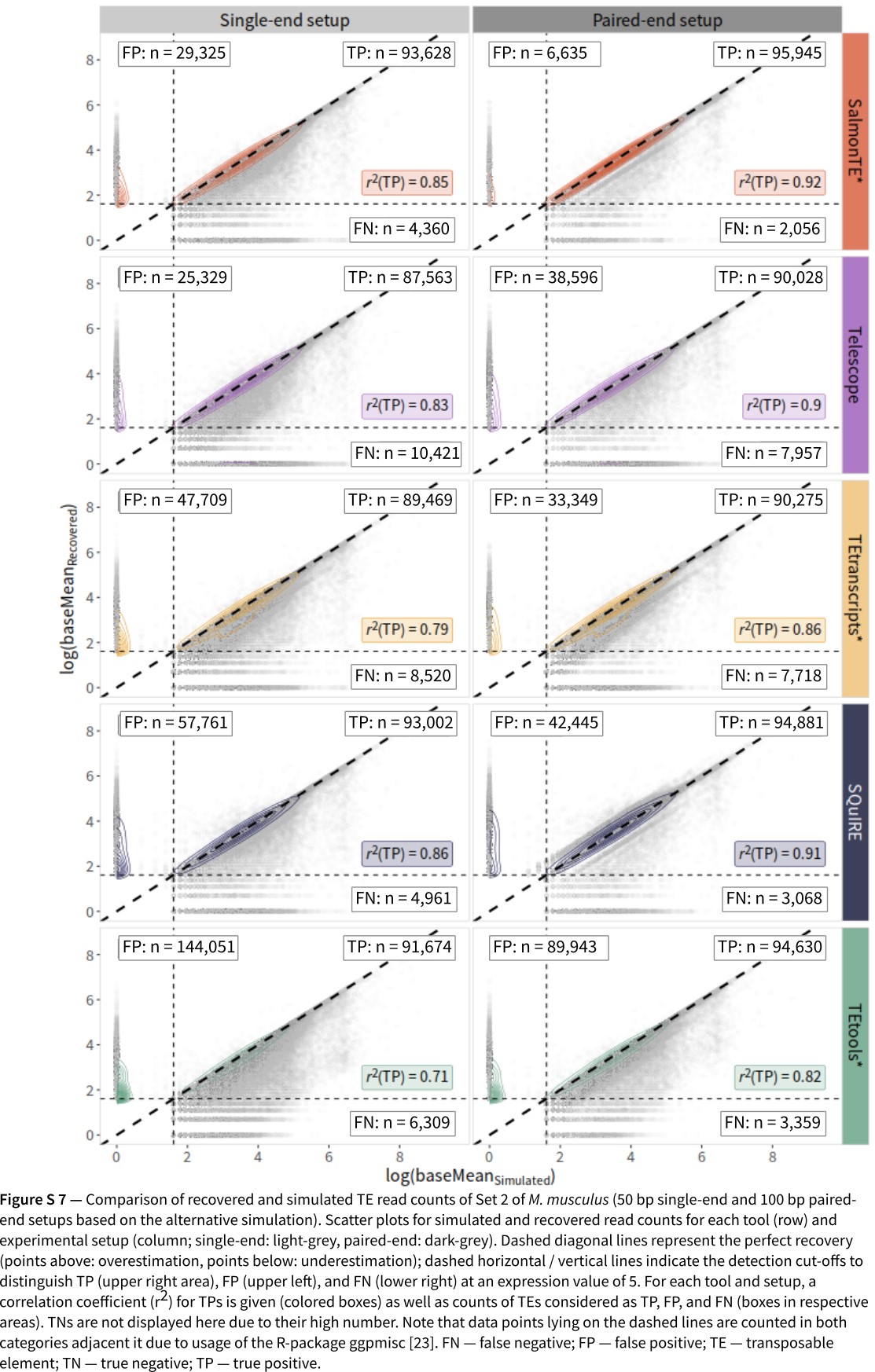

Supplement: figure_S7_bbab417 [file figure_s7_bbab417.jpeg]

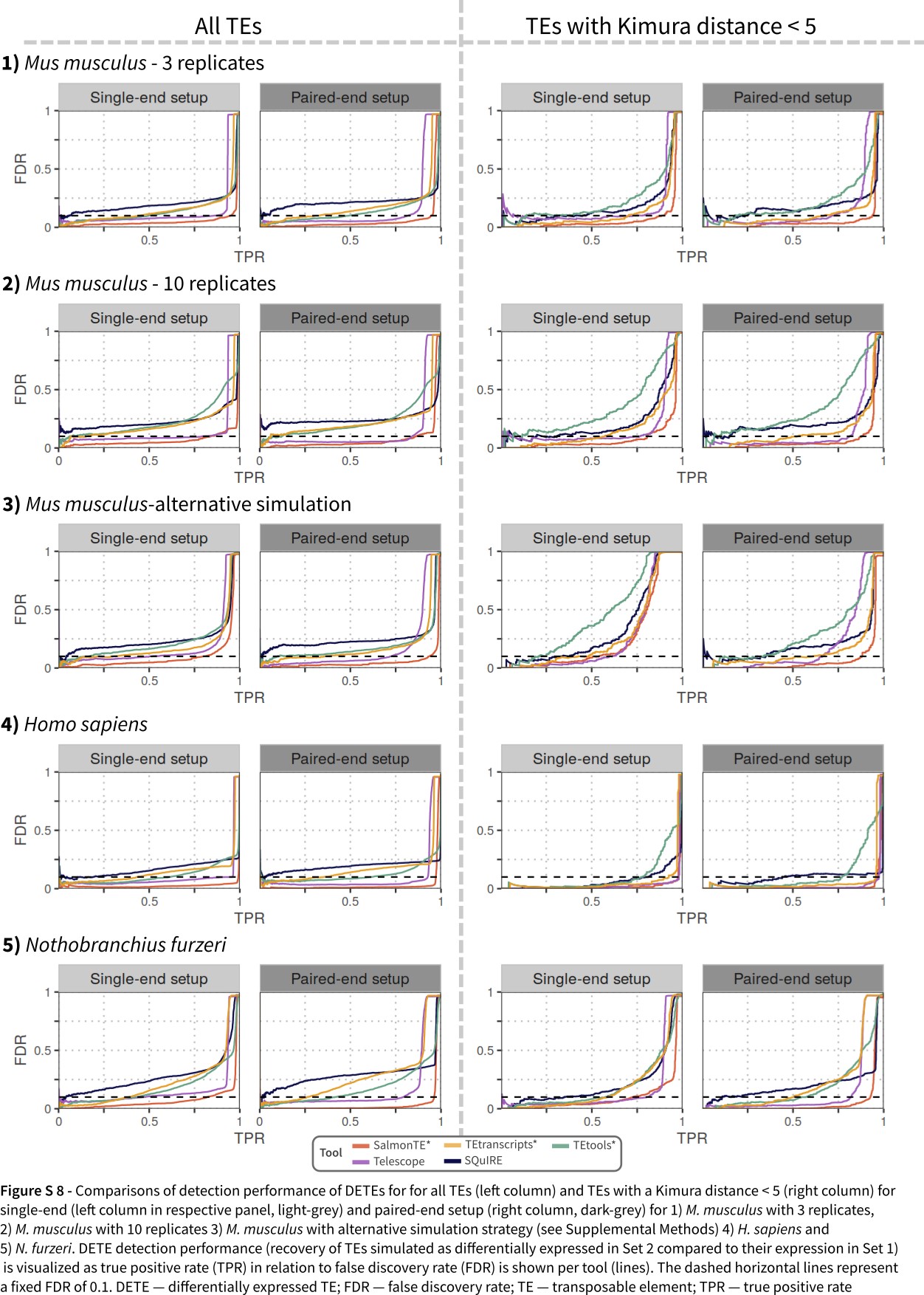

Supplement: figure_S8_bbab417 [file figure_s8_bbab417.jpeg]
