# Supplementary material for: Locus-specific expression analysis of transposable elements
Source: Brief Bioinform. 2021 Oct 19;23(1):bbab417. doi: 10.1093/bib/bbab417 (PMC8769692; doi:10.1093/bib/bbab417)
Supplement: Supplemental_File_6_bbab417 [file supplemental_file_6_bbab417.pdf]

Supplemental File 6 of Locus-specific expression analysis of transposable elements

Contents

Overview **2**

Preparation **3**
Generation of reference library . . . 3

Simulation **3**
Polyester . . . 3
Select a random set of 100.000 TEs . . . 3
Simulation of reads . . . 4
Alternative simulation . . . 4
Select a random set of 100.000 TE instances . . . 4
Simulation of reads . . . 5

Run Tools **6**
Preparation of tool-specific files . . . 6
Creation of index for SalmonTE . . . 6
Create rosette file for TEtools . . . 6
Creation of Clean directory and dictionary for SQuIRE . . . 6
Create alignment files with STAR for TEtranscripts* and Telescope . . . 6
Apply Tools . . . 7
SalmonTE* . . . 7
Telescope . . . 7
TEtools* . . . 8
TEtranscripts* . . . 8
SQuIRE . . . 9

Evaluation **9**

Overview

In the study "Locus-specific expression analysis of transposable elements" the performances of SalmonTE*, SQuIRE, Telescope, TEtools* and TEtranscripts* were evaluated with respect to the detection and quantification of transposable elements (TEs) and the detection of differentially expressed TEs. This document is structured by analysis steps and lists all tools used in the respective step with their purpose, used arguments, and the argument descriptions (partially copied from the reference of the respective tool). All scripts that are not listed within Table 1 are in-house script, which can be found at GitHub:

https://github.com/Hoffmann-Lab/TEdetectionEvaluation

The following tools were used for this study:

Table 1. Tools that were used in this study.

Tool	Version	Resource
bedtools	2.29.2-41-g4ebba703	https://bedtools.readthedocs.io/en/latest/
Bioconductor	3.10	https://bioconductor.org/about/
DESeq2	1.26.0	https://bioconductor.org/packages/release/bioc/html/DESeq2.html
Polyester	1.22.0	https://github.com/alyssafrazee/polyester
Salmon	0.9.1	https://salmon.readthedocs.io/en/latest/
SalmonTE	0.4	https://github.com/LiuzLab/SalmonTE
SQuIRE	0.9.9.92	https://github.com/wyang17/SQuIRE
STAR	2.7.6a	https://github.com/alexdobin/STAR
Telescope	1.0.3	https://github.com/mlbendall/telescope
TEtools	1.0.0	https://github.com/douglasgscofield/TEtools
TEtranscripts	2.2.1	https://github.com/mhammell-laboratory/TEtranscripts

The following references were used for this study:

Table 2. References that were used in this study. The entries of the Resource column are hyperlinks.

Reference	Build	Version	Resource
H. sapiens genome assembly	hg38	102	Storage of assembly
M. musculus genome assembly	mm10	102	Storage of assembly
N. furzeri genome assembly	Nfu_2015022	05/2015	Storage of assembly
H. sapiens TE annotation	hg38	db20140131	http://www.repeatmasker.org/species/hg.html
M. musculus TE annotation	mm10	20140131	http://www.repeatmasker.org/species/mm.html
N. furzeri TE annotation	Nfu_2015022	05/2015	https://nfingb.leibniz-fli.de/

Preparation

Generation of reference library

A reference library (FASTA-format) of the annotated TEs (contained in the .align-files) was created. The in-house script `align_parser.py` was used to extract the coordinates of the annotated TEs in a BED-format with the following call:

```
align_parser.py -a <.align-file>
```

Table 3. Overview of arguments of `align_parser.py`

Argument	Description
-a	alignments generated by RepeatMasker

The reference sequences were extracted with `bedtools` and stored as FASTA-format (`referencelibrary.fa`) with the following command:

```
bedtools getfasta -fi <reference-genome> -bed <.bed-file> > referencelibrary.fa
```

Table 4. Overview of arguments of `bedtools getfasta`

Argument	Description
-fi	reference genome in fasta format
-bed	TE annotation in bed format

Simulation

Polyester

Select a random set of 100.000 TEs

The script `sampleTEs.R` was used to randomly select 100.000 TEs for the polyester simulation with the following call:

```
Rscript sampleTEs.R -m <int> \
  -s <organism> \
  -c <int> \
  -b <bed-file> \
  -f <name of FASTA file> \
  -o <name of result direcory>
```

Table 5. Overview of arguments of `sampleTEs.R`

Argument	Description
-m	minimal length of TE
-s	species
-c	number of TEs that is extracted
-b	.bed-file that containes the coordinates of TEs
-f	.fasta-file with the TE reference sequences

Argument	Description
-o	name of the result directory

For this study following parameters are selected:

- minimal length of TE is set to 100
- species is set to human mouse or notho as required
- number of TEs is set to 100.000
- .bed-file & .fasta-file is set respected to the file names

Simulation of reads

The tool polyester was used to simulate reads for the main part of the study. The polyester call was implemented in the in-house script `simulation_polyester.R` and called as follows:

```
Rscript simulation_polyester.R -d <int> \
  -f <name of FASTA file> \
  -r <int> \
  -l <int> \
  -o <output dir> \
  -s <single/paired>
```

Table 6. Overview of arguments of `simulation_polyester.R`

Argument	Description
-d	percentage of differentially expressed TEs
-f	fasta file that contains reference sequences
-r	number of replicates per group
-l	defines the read length (default: 100)
-o	name of output directory
-s	defines if a single- or paired-end data set is simulated

For this study following parameters are selected:

- percentage of differentially expressed TEs (-d) is set to 5
- number of replicates (-r) is set to 3, 5 or 10 as required
- read length (-l) is set to 50 or 100 as required
- setup (-s) is set tot paired or single as required

Alternative simulation

Select a random set of 100.000 TE instances

```
getRandomSeq -f <name of FASTA file> \
  -n <int>
  -l <int>
  -o <name of output directory>
  -s <int>
```

Table 7. Overview of arguments of `getRandomSeq`

Argument	Description
-f	fasta file that contains reference sequences
-n	number of TEs that is extracted
-l	minimal length of TE
-o	name of output directory
s	seed for reproducibility

For this study following parameters are selected:

- minimal length (-l) of TE is set to 100
- number of TEs (-n) is set to 100.000
- seed (-s) is set to 5

Simulation of reads

The alternative simulation, which is contained in the study, was done with the in-house script `readiator` and was called as follows:

```
readiator -f <name of FASTA file> \
  -fq <name of FASTQ file> \
  -l <int> \
  -o <name output directory> \
  -r <int> \
  -sz <int> \
  -s <int> \
  [-d] [-p]
```

Table 8. Overview of arguments of `readiator`

Argument	Description
-f	fasta file that contains reference sequences
-fq	a fastq file from a real experiment
-l	defines the read length
-o	name of output directory
-r	number of reads per simulated fastq file
-sz	number of replicates per group
s	seed for reproducibility
-d	simulation of a second group with differentially expressed TEs (optional)
-p	simulation of paired end data (optional)

For this study following parameters are selected:

- simulation of a second group with differentially expressed TEs (-d)
- read length (-l) is set to 50 or 100 as required
- desired number of reads (-r) is set to 5.000.000
- sample size per group (-sz) is set to 5
- seed (-s) is set to 5

- simulated a paired-end data set as required (-d)

Run Tools

Preparation of tool-specific files

Files that are needed by the respective tools were prepared as listed within this section.

Creation of index for SalmonTE

```
salmon index -t <name of FASTA file> -i <index name> -type quasi -k 31
```

Table 9. Overview of arguments of `salmon`

Argument	Description
-f	reference sequences of TEs in fasta format
i	name of the index
-type	type of index to build
k	size of k-mers used for the quasi index

Create rosette file for TEtools

```
awk {'print $4"\t"$4'} <repeat annotation in .bed format>
```

Creation of Clean directory and dictionary for SQuIRE

```
# translate the .align file to a .out file
align_to_out.py <.align-file> | sort | uniq > <.out-file>

# Use squire Clean to generate a .bed-file that is needed by SQuIRE
squire Clean -r <out-file> -b <name of build>

# generate dictionary to translate TE ids in down-stream analysis
generateDict.py squire_clean/<.bed-file> <.bed-file reference library>
```

Create alignment files with STAR for TEtranscripts* and Telescope

The alignment files generated by STAR serve as input for TEtranscripts* and Telescope.

```
STAR --genomeDir <index of genome> \
  -- readFilesIn <fastq-file> \
  --winAnchorMultimapNmax <int> \
  --outFilterMultimapNmax <int> \
  --alignIntronMax <int> \
  --outFilterMismatchNoverLmax <float>
```

The simulated sequencing data was aligned with STAR (v2.7.6a) according to the recommendation of the authors of TEtranscripts with the following options:

- winAnchorMultimapNmax is set to 100

Table 10. Overview of arguments of STAR

Argument	Description
genomeDir	specifies path to the directory where the genome indices are stored
readFilesIn	list of fastq files
winAnchorMultimapNmax	max number of loci anchors are allowed to map to
outFilterMultimapNmax	max number of multiple alignments allowed for a read
alignIntronMax	maximum intron length
outFilterMismatchNoverLmax	alignment will be output only if its ratio of mismatches to *mapped*length is less than or equal to this value

- outFilterMultimapNmax is set to 100
- alignIntronMax is set to 100000
- outFilterMismatchNoverLmax is set to 0.04

Apply Tools

The tool calls, which were used to estimate counts of TEs, are listed within this section.

SalmonTE*

```
SalmonTE.py quant --reference=<index name> \
    --outpath=<name output dir> \
    --exprtype=count \
    <list of fastq files>
```

Table 11. Overview of arguments of `SalmonTE`

Argument	Description
reference	name of the generated index
outpath	name of result directory
exprtype	type of the expression measure

Telescope

```
telescope assign <alignment file> \
    <.gtf-file of TEs> \
    --attribute gene_id \
    --outdir <name output dir>
```

Table 12. Overview of arguments of `Telescope`

Argument	Description
alignment file	alignment file in bam format
gtf-file	gtf file with the TE annotation
attribute	GTF attribute that defines a transposable element locus.
outdir	name of result directory

TEtools*

```
TEtools -rosette <rossette file> \
    -TE_fasta <name of FASTA file> \
    -count <name of result file> \
    -RNA <list of fastq files>
```

Table 13. Overview of arguments `TEtools`

Argument	Description
rosette	name of rosette file
TE_fasta	TE reference sequences in FASTA format
count	name of the count table
RNA	list of FASTQ files

TEtranscripts*

```
TEtranscripts --sortByPos \
    --format BAM \
    --mode multi \
    --project <name of result dir> \
    --GTF <.gtf-file of geness> \
    --TE_GTF <.gtf-file of TEs> \
    -t <fastq files DETEs> \
    -c <fastq files of control>
```

Table 14. Overview of arguments of `TEtranscripts`

Argument	Description
sortByPos	define if the alignment files are sorted
format	type of the alignment file
mode multi	distributes multi-mapping reads
project	name of the output directory
GTF	Gene annotation in GTF format
TE_GTF	TE annotation in GTF format
-t	list of fastq files with differentially expressed TEs
-c	list of control fastq files

SQuIRE

The shipped bash-scripts from SQuIRE were used. The following command is copied from the bash-script, which is responsible for the read count. The creation of the clean_folder is named in the 'Generate tool specific files' section of this document.

```
squire Count \
    --map_folder <map_folder> \
    --clean_folder <clean_folder> \
    --count_folder <count_folder> \
    --temp_folder <temp_folder> \
    --name <name of result dir> \
    --build <name of build>\
    --strandedness 0 \
    --EM auto \
    --read_length <read length> \
    --fetch_folder <fetch_folder>
```

Table 15. Overview of arguments of `squire Count`

Argument	Description
map_folder	Folder location of outputs from SQuIRE Map
clean_folder	Folder location of outputs from SQuIRE Clean
count_folder	Name of the output directory
temp_folder	Folder for tempfiles
name	Common basename for input files
build	Name of the build you want to use, e.g. mm10
strandedness	0 if unstranded eg Standard Illumina
EM	Run estimation-maximization on TE counts given numberof times
read_length	Read length
fetch_folder	Folder location of outputs from SQuIRE Fetch

Evaluation

After adapting the `general.R` and `dataInfo.csv` files run the evaluation script as follows:

```
Rscript TEdetectEval.R
```

Subsequently, the scripts `figure.R` and `table.R` need to be run to generate figures and tables.
