# Supplementary material for: Locus-specific expression analysis of transposable elements
Source: Brief Bioinform. 2021 Oct 19;23(1):bbab417. doi: 10.1093/bib/bbab417 (PMC8769692; doi:10.1093/bib/bbab417)
Supplement: 2021-05-20_Supplemental_Methods_bbab417 [file 2021-05-20_supplemental_methods_bbab417.docx]

**Supplement Methods**

*Simulation of short read RNA-Seq data with polyester*

The simulation was done by two steps (i) Simulation with all parameters as named in the methods part, without a GC bias (ii) Usage of the count table from the step (i) apply the GC-Bias simulation to the count table and run the final simulation with the updated count table. Since, polyester cannot handle 0 in the count table, but those were introduced by the GC-Bias simulation, we added a pseudo count of 1 to each TE and sample. Pseudo-quality scores were added to the simulated FASTA files with the script ‘reformat.sh’ from the BBMap-package^[[1]](#footnote-1)^. The source code of this simulation can be found in more detail at GitHub: <https://github.com/Hoffmann-Lab/TEdetectionEvaluation>/[simulation_polyester.R](https://github.com/roschwarz/TEdetectionEvaluation/blob/main/simulation_polyester.R).

*Alternative simulation of short read RNA-Seq data*

In the alternative simulation strategy, we consider single-end (50 bp read length) as well as paired-end sequencing (100 bp read length) experiments. For either experimental setup, two distinct sets with 5 biological replicates each are generated. Throughout this study, the first set is considered a control (Set 1) while the second set contains 5% uniformly randomly drawn DETEs (Set 2). As a basis for our simulation, 100,000 TEs with at least 100 bp in length and a known Kimura distance are uniformly randomly drawn from the reference library. To make the simulations more realistic, quality strings of real sequencing runs (GEO accession numbers for single-end setup: GSM1944865^[[2]](#footnote-2)^, paired-end setup: GSM1716945^[[3]](#footnote-3)^) were used to systematically introduce sequencing errors resembling the error profile of the Illumina HiSeq 2500 (single-end) and HiSeq 2000 (paired-end) sequencing platforms. More specifically, data is generated in three consecutive steps.

Step 1: First, 5% of 100,000 selected TEs are randomly flagged as differentially expressed. For each flagged TE, fold-changes are determined by uniformly drawing a direction (up-/down-regulation) and a factor of the interval 2 to 10. Direction and factor provide the basis for the generation of counts in Set 2.

Step 2: For each TE in each set, a read count base is generated involving draws of random numbers from Dirichlet and negative binomial (NB) distributions. First, we are interested in simulating relative expression rates for all TEs. To achieve this, the length of each TE is divided by the minimum TE length (100 bp), rounded up to the next integer and the values are summed up across all TEs to obtain a total tile count, *k*. For all *k* tiles, random real-valued numbers are drawn from a Dirichlet distribution ensuring that all values sum up to 1. Subsequently, values for tiles belonging to the same TE are aggregated to obtain the desired relative expression rate for each TE. To obtain mean expression levels for the TEs, expression rates are multiplied by the desired number of reads (5,000,000). For flagged TEs in Set 2, mean expression levels are additionally multiplied by the factor and direction determined in Step 1. The generated mean expression levels serve as input for the NB distribution to obtain expression values (read counts) for each TE $i$ of each sample$j$, $n_{ij}$(Read counts $\in K^{\mathrm{ixj}}$). These counts are stored in a table that serves as reference for the tool evaluation.

Step 3: In the final step, we simulate the sequencing process by introducing Illumina HiSeq 2000 and 2500-specific error profiles. Specifically, for each sample and each TE, $n_{ij}$, reads uniformly distributed across the TE sequence are drawn. The drawn reads are stored as reverse-complemented with a probability of 0.5 for the single-end setup. For the paired-end setup, fragment lengths are determined by a normal distribution with mean length 200 bp and a standard deviation of 100. Subsequently, reads are extracted from the 5’ and 3’ ends of each fragment and the 3’-read is reverse complemented. All instances that received less than five reads in sum across all samples are excluded from the analysis. Finally, the Illumina-specific error profiles are introduced by using phred-scores obtained from real sequencing run. Based on the phred-scores, individual bases are altered to one of the other three nucleotides. In case of ambiguous bases (N), a change to A, T, G, C, and N is allowed. Results for each sample and setup are stored in FASTQ files.

The source code for the simulation can be found on <https://github.com/Hoffmann-Lab/readiator>.

*Ranking*

The tools were ranked for three different exercises: Detection of TE expression, quantification of TE expression and, detection of differential TE expression. Therefore, the tools get points in different exercise-specific categories (5 points for best to 1 point for worst performance). The points were summed up per exercise to calculate exercise-specific ranks. All species and sequencing approaches were considered for the exercise-specific ranks. The same ranking procedure was separately done for the alternative simulation strategy.

1. B., B. BBMap https://sourceforge.net/projects/bbmap/. 2014. [↑](#footnote-ref-1)
2. Huhne, R., Thalheim, T. and Suhnel, J. AgeFactDB--the JenAge Ageing Factor Database--towards data integration in ageing research. *Nucleic Acids Res* 2014;42(Database issue):D892-896. [↑](#footnote-ref-2)
3. Li, X.*, et al.* Whole-genome analysis of the methylome and hydroxymethylome in normal and malignant lung and liver. *Genome Res* 2016;26(12):1730-1741. [↑](#footnote-ref-3)
